# Supplementary material for: The Royal Zoological Society of Scotland’s Approach to Assessing and Promoting Animal Welfare in Collaboration with Universities
Source: Animals (Basel). 2024 Jul 31;14(15):2223. doi: 10.3390/ani14152223 (PMC11311029; doi:10.3390/ani14152223)
Supplement: Supplementary file 1 [file animals-14-02223-s001.zip › Figure S1_Systematic behavioural sampling to gain estimates.pdf]

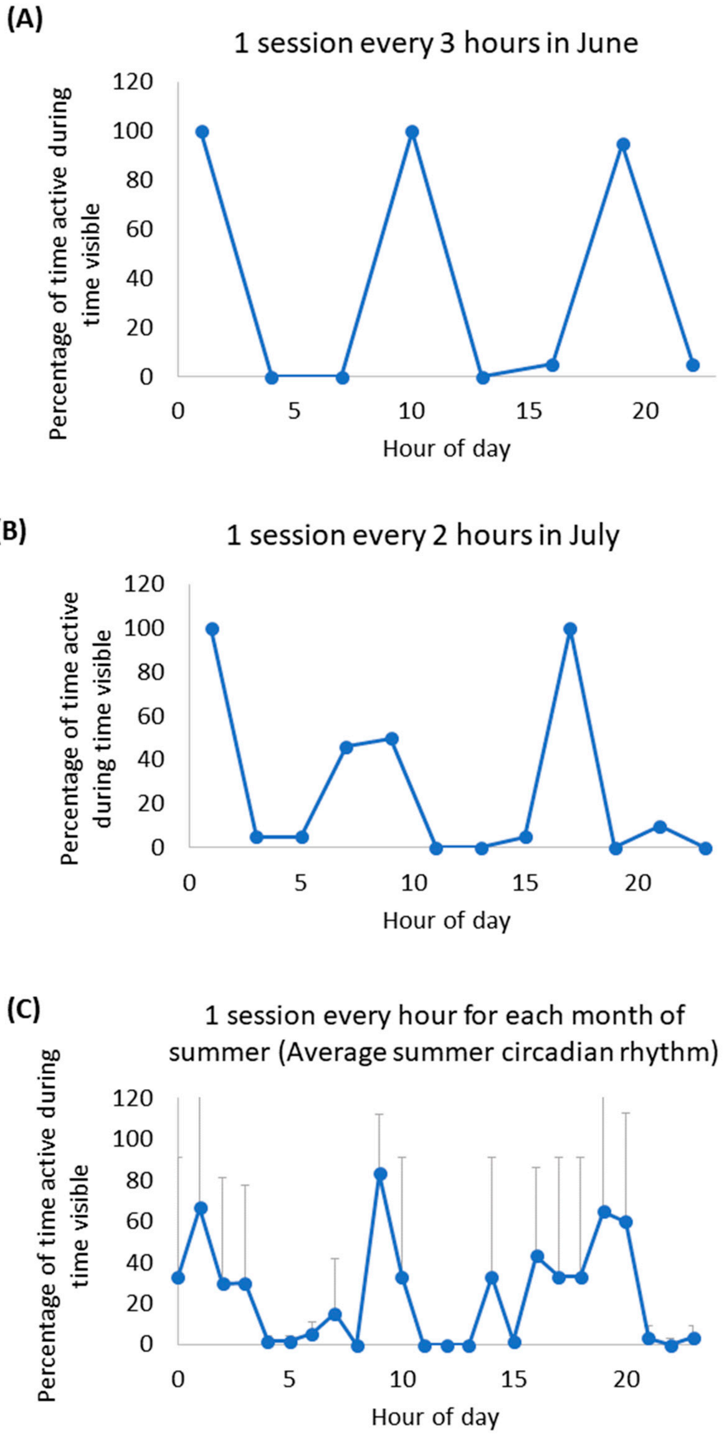

Figure S1. Adopted from Gandia [104]. Gandia [104] used an identically structured ethogram to those used in this study which was converted to a ZooMonitor project to gain estimates of activity cycles in giant pandas. This figure demonstrates how few sessions are needed to gain rough estimates of these cycles when the sessions are conducted systematically. The following is the original caption in Gandia [104]: Depictions of circadian rhythm estimates of activity (any behaviour that is not resting/sleeping)

over a Summer season for one giant panda (sub-adult female) using increasingly more sessions (each session is 10 minutes with 20 intervals) to form an estimate. (A) Estimate using 8 sessions total, systematically collected every 3 hours in the month of June. (B) Estimate using 12 sessions total, systematically collected every 2 hours in the month of July. (C) Estimate using 72 sessions total, with 24 sessions collected systematically every hour for each month of Summer (June, July, and August). The grey bars are standard deviations (positive value only) in C. The progression in these estimates illustrates how a decent estimate can be gained by systematically collecting data to span evenly across a full 24-hr period. The three peaks in activity can be seen in all three graphs, demonstrating how important patterns can still be captured with less detailed data.
