# Supplementary material for: The Royal Zoological Society of Scotland’s Approach to Assessing and Promoting Animal Welfare in Collaboration with Universities
Source: Animals (Basel). 2024 Jul 31;14(15):2223. doi: 10.3390/ani14152223 (PMC11311029; doi:10.3390/ani14152223)
Supplement: Supplementary file 1 [file animals-14-02223-s001.zip › Table S1_Studies conducted at RZSS related to Five Domains.pdf]

Table S1. A summary of selected studies conducted at RZSS Edinburgh Zoo since 1996, split into Five Domains categories, and the key findings in relation to animal welfare.

Five Domains category	Main findings of research in relation to welfare (reference at end)
**Nutrition**	The rates of regurgitation and reingestion (R&R, that may have serious health negative health consequences [131]) in the chimpanzees *(Pan troglodytes)* were higher within 40 minutes of a feed, but not affected by the inter-feed interval preceding that feed. Positive or negative social interactions, or visitor numbers or behaviour did not affect the R&R rate. Dietary change did not affect R&R rates either suggestion that nutritional composition of the diet was not the key trigger. [48]
	Wood-pile feeders increase the searching time in captive bush dogs (*Speothos venaticos*), potentially improving welfare, although searching time declined over time with likely increased proficiency of finding food. [49]
	The behaviour of nine felid species was influenced by feeding regime. Stereotypic pacing levels with felids fed on a 3-day cycle paced more on fast days than on days they were fed. [50]
	For species adapted to hunt, an inability to hunt may reduce their welfare. Two captive cheetahs *(Acinonyx jubatus) w*ere provided with moving bait which increased the frequency of sprinting (hunting) and decreased time spent in affiliation and feeding - effects which were observed even when the moving bait was absent, suggesting their welfare may be improved [51]
	Additional foraging opportunities appropriate for parrots showed that they increased allopreening compared to baseline or post-enrichment periods. There was also evidence of contra freeloading (a preference to work for food than to access food freely available) but no preference for a variable over a constant food source. [52]
	Using carnivores as an example, the research highlights that feeding captive animals is more complex than offering a nutritionally balanced diet. In addition to nutrition, food should be offered in an appropriate manner, considering the reasons why that animal is kept in captivity in terms of the four objectives of modern zoos (conservation, education, research and entertainment) and animal welfare. [53]
**Physical Environment**	Using Social Network Analyses (SNA), the data show that the Budongo Trail exhibit in Edinburgh Zoo, with its interconnected "pods" allows chimpanzees to exhibit social preferences, likely improving welfare, and SNA as a tool may assist zoo staff in their management decisions. [54]
	Enclosure space is important to captive chimpanzees, but during introductions, a decrease in arousal-related scratching indicates that the number of accessible areas is more important than the total amount of space available. This suggests that providing modular enclosures that provide choice and flexible usage is important to minimise the welfare impact of short- and long-term husbandry needs. [55]
	Giant pandas (*Ailuropoda melanoleuca*) are known to become stressed when subjected to undesirable noise which may seem unobtrusive to humans, as pandas have a broader hearing range than humans. This paper discusses the adaptation from international standards on noise in relation to construction site noise on giant pandas. [56]
	Chimpanzee behaviour was compared when silence, classical or pop/rock music was played in one of several indoor areas. The chimpanzees did not actively avoid the area when music was

	playing but exited the area more often when songs with higher beats per minute were playing. They exhibited fewer active social behaviours when music, rather than silence, was playing and were more active and engaged in less abnormal behaviour during the music than with silence. Neither self-grooming nor aggression were affected. When given a choice to listen to music of various types or silence there were no persistent preferences for any type of music or silence. The results do not suggest that music is enriching for group-housed captive chimpanzees, nor that it has negative effects on welfare if the chimpanzees can choose to avoid it. [57]
	Four species of guenon were studied, and their responsiveness to objects that represented high and low levels of both visual complexity and responsiveness were recorded. The overall finding is that captive monkeys prefer responsive objects to unresponsive ones but have no preference for visual complexity over simplicity, with implications for choice of enrichment items. [58]
	Reliably signalling a startling husbandry event (keeper entrance to the enclosure) improves welfare of capuchins (*Sapajus apella*). [59]
	Neither daytime associations nor presence of kin influenced sleeping site selection in female chimpanzees, but in males' daytime associations influenced sleeping arrangements. The nighttime sleeping arrangements were similar to nesting patterns reported in wild chimpanzees. To promote captive ape welfare, it is suggested that exhibits should incorporate multilevel nesting areas and choices of different sleeping sites. [60]
**Health**	The RZSS were key partners on veterinary health surveillance on the Scottish Beaver Trial, where 16 wild Norwegian beavers (*Castor fiber*) were translocated and released in Scotland. [61]
	The RZSS in collaboration with other conservation bodies identified the true pregnancy length of the giant panda (*Ailuropoda melanoleuca*) and other health indicators that could be gathered in a non-invasive manner. [44]
	The RZSS identified a novel testing modality to detect aspergillosis a potentially lethal environmental fungal respiratory disease in captive gentoo penguins (*Pygoscelis papua*), that would allow early detection of disease and so increase treatment success and reduce mortality levels. [43]
	The RZSS produced the first peer-reviewed and published disease risk analysis for reintroducing the Eurasian beaver to Great Britain identifying the important risk factors for beavers, wildlife, domestic animals and humans. Girling et al. [41] also identified that the Eurasian beaver was not a significant concern for *Leptospira* spp. infection spread for the first time. [41]
	The RZSS produced a treatment regimen that reduced first year mortality rates of captive bred Pallas' cats (*Otocolobus manul*) in Europe by 67%. [42]
	The RZSS reviewed, critically evaluated and identified the significant causes of mortality and morbidity in captive UK populations of European wildcats over a 21-year period from 2000-2021 as part of the Saving Wildcats programme. [39]
	An alternative method of administering oral antibiotics for bacterial airsacculitis was developed via the use of a nebulizer when the chimpanzee developed resistance to all available oral preparations. The infection was successfully treated and the research describes how positive reinforcement training is a useful tool in the successful welfare and management of captive animals. [37]
	Description of fatal cowpox virus infection in two squirrel monkeys (*Saimiri sciureus*) with implications for identifying factors influencing the progression of cowpox in captive non-human primates to attempt to prevent (or limit) future outbreaks. [132]

	A review and recommendations of the tiger feeding pole indicating a range of benefits of use especially that the skeletons of feeding-pole-using tigers had reduced arthrosis scores compared to tigers that did not use poles, a positive indicator of the health benefit of the use of this enrichment device. [62]
**Behavioural Interactions**	With a focus on primates, this paper concludes that zoos should strive to carry out environmental enrichment, providing an interesting, stimulating and varying environment which in turn, will enable zoos to fulfil their conservation, research, education and entertainment aims. [63]
	A comparison of different properties of "replica fruits" in Barbary and stump-tailed macaques showed that replica fruits were manipulated most when they functioned as foraging devices. It is concluded that objects that increased the animals' sense of control in addition to providing food rewards appeared particularly suitable as enrichment devices. [65]
	The programme of enrichment for birds is described from the planning of aviaries to the time allocated in the daily routine by incorporating enrichment techniques into the basic husbandry procedures. [133]
	Capuchin monkeys with similar personalities have higher-quality relationships independent of age, sex, kinship and rank. Understanding personality may be used in management decisions aiming to promote welfare. [66]
	This methodological paper demonstrates that staff ratings of chimpanzee personality and subjective wellbeing were consistent between observers and over time. Chimpanzees with positive welfare are rated as happier, extraverted, and emotionally stable. It is concluded that questionnaires completed by keepers or others familiar with chimpanzees are a reliable and valid tool for assessing chimpanzee welfare. [67]
	The process of successfully integrating two groups of chimpanzees in the Budongo Trail facility Edinburgh Zoo was documented using social network analysis over the 16 months following integration. Aggression rates were low and decreased over time whilst positive relationships between members of the original groups became stronger and more affiliative. However, two distinct subgroups were still evident a year after integration. It is concluded that when freedom to exhibit natural fission–fusion groupings in complex space strong affiliative relationships with unfamiliar individuals can develop over time. [68]
	Mixed species exhibits have several benefits for individual animals but might also create welfare problems such as stress from interspecific aggression. The behaviour of single and mixed species groups capuchin monkeys (*Sapajus apella*) and squirrel monkeys (*Saimiri sciureus*) which naturally associating in the wild was recorded in Edinburgh Zoo. We found no evidence of chronic stress in the subordinate squirrel monkeys who actively chose to associate with the capuchins. Modifications to the enclosure were successful in reducing the occasional mildly aggressive interactions with affiliative interactions increasing in frequency and diversity. The findings suggest that in carefully designed, large enclosures, with regular monitoring of inhabitants and enclosure modifications as required, naturally associating monkeys can live harmoniously and are enriched by each other. [38]
	This is an extension of the Leonardi et al. [38] paper describing how interspecific interactions and welfare changed over 3 years, demonstrating the need to assess welfare over time. [72]
	The social network, daily activity and the expression of stress-related behaviour in capuchins (*Cebus apella*) and squirrel monkeys (*Saimiri sciureus*) was recorded before and just after they were relocated to a new enriched enclosure. Both species increased the time spent resting,

	spent more time in the highest and "safest" part of their enclosure, and more time in close proximity to conspecifics. Social network analysis provided a more complete picture of how individuals were affected by relocation and may help management decisions in times of stressful events such as relocation to attempt to improve welfare. [71]
	This study examined space use in two mixed-species groups of capuchins and squirrel monkeys. Capuchins preferred the centre of the enclosure while squirrel monkeys spent more time in their exclusive indoor enclosure and spent more time in peripheral zones of their outdoor enclosures and close to doorways leading indoors. The data suggest that housing these species in a mixed exhibit may not be cognitively enriching but that it does provide appropriate cognitive challenges that can still enhance the welfare of individuals. [73]
	Social Network Analyses is applied to two captive mixed species populations of capuchins and squirrel monkeys. Network diagrams for both mixed species groups (MSGs) showed distinct clusters, separating the species. Factors such as relative dominance and individual/group differences also affected interaction patterns. The findings suggest that, in captivity at least, while these groups co-exist in a small, shared space, they do not form interactive MSGs highlighting the need for careful enclosure design and monitoring. Daoudi-Simison et al submitted
	Goeldi's monkeys (*Callimico goeldii)* and pygmy marmosets (*Cebuella pygmaea)* are sympatric in nature and were housed together in a mixed-exhibit. Pygmy marmosets were submissive to Goeldi's monkeys, but no physical interactions were observed. The two species used enclosure space differently, and in a similar way to the wild which may contribute to their peaceful co-existence. [69]
	The individual personalities in a colony of three species of penguins living in the same enclosure had some common personality traits across species, with some expressed with a different intensity. The findings can inform housing and husbandry decisions, including enclosure design and environmental enrichment in relation to the different individual and species characteristics. [70]
	Marabou stork (*Leptoptilos crumeniferus*) can be aggressive in captivity, and require careful management during introductions, and a sound understanding of behaviour especially when choosing individuals to house together. Their feet need to be closely monitored due to their susceptibility to foot problems. Positive reinforcement training eased the movement of birds, reduced aggression and allowed regular health and feet check in a low-stress manner. Such husbandry training is effective, does not require excessive time or staff costs, and may be rewarding for both birds and trainers. [74]
	A study exploring visitor density and visitor eating behaviour found that primates were affected by high numbers of visitors eating bananas. The primates spent more time looking at visitors who were eating. This research is important as it may affect the placement of picnic sites. [32]
