# Supplementary material for: The Royal Zoological Society of Scotland’s Approach to Assessing and Promoting Animal Welfare in Collaboration with Universities
Source: Animals (Basel). 2024 Jul 31;14(15):2223. doi: 10.3390/ani14152223 (PMC11311029; doi:10.3390/ani14152223)
Supplement: Supplementary file 1 [file animals-14-02223-s001.zip › Table S2_RZSS Welfare Assessment Tool.pdf]

Table S2. The current RZSS Welfare Assessment Tool. The current guidance provided to assessors when completing the welfare assessment is included. The table displays the questions and the current guidance on how to respond to the question as well as the suggested evidence-bases that are being developed and piloted. In addition, certain questions are being reworded to diminish ambiguity or provide more clarity on exactly what the question is asking (e.g. questions 2, 10, 12, 14, 15, 16 and any other questions we find assessors have difficulty interpreting). For behaviour questions, as ethograms for taxa are developed, we will also suggest that at a minimum (should ZooMonitor observations not be possible), assessors consult the ethogram to understand the behavioural repertoire of the species and the behaviours which pertain to each question.

Animal Welfare Assessment Guidance (as of July 2024)

AIMS OF AN ANIMAL WELFARE AUDIT

- To identify any current immediate welfare problems.
- To identify if information pertinent to assessing welfare is absent or difficult to access.
- To provide a structure frame work to quantify changes in welfare over time.

Our welfare assessment has been put together using the BIAZA Animal Welfare Toolkit

ASSESSORS

Ideally more than one person will carry out each assessment independently:

- Keepers
- Vets
- Living Collection Dept managers
- Suitably qualified and trained volunteers

are all suitable assessors.

ASSESSMENT TARGETS

Each assessment can cover:

- a single animal
- a species of animals within a single enclosure or
- a mixed species group of animals in a single enclosure.

This assessment is designed to cover all vertebrate animals.

EVIDENCE

In general terms the assessment will consider both:

- RESOURCE-BASED indicators of welfare (what resources does the animal have available to it?) and
- ANIMAL-BASED indicators of welfare (behavioural/physical/physiological signs exhibited by the animal)

These indicators are used as evidence to assess the animals likely resultant mental state.

- Assessors must visit the exhibit and make direct observations of the animals in the current moment

Assessors may also make use of (for example):

- Best practice guidelines
- Diet sheets, feeding, weight and body condition records
- ZIMS husbandry and veterinary records
- Enrichment and training records

Some question may require both direct observation and remote research to fully answer, other may rely on one or the other. Use the check boxes to indicate evidence type, or document in the notes section.

SCORING

The 50 questions are now arranged into 4 sections to make completing a welfare assessment more efficient and less time consuming. Each section can be answered in any order and does not need to be completed on the same day.

Each of 50 questions can be answered as follows:

Yes: the statement is true for all individuals in the enclosure

No: the statement is not true for at least one of the individuals in the exhibit – include details of which animals, and in what way the criterion has been failed in the notes and if there any mitigations already in place

N/A: Not applicable – not relevant to the species/individuals being assessed

Unknown: the assessor could not make a judgement – this might be because the evidence is not there, because there is no way to assess that criteria currently, because the subject matter was outside of the assessors expertise or because the evidence couldn't be accessed on the day. The assessor should note why they could not make a judgement.

Write as many notes as you would like/have time for!

NEXT STEPS

Each assessor should return their audit to the RZSS Curator for collation. If you use the on-line form this is automatic.

Assessors should meet to discuss their scores and to agree a final audit outcome.

The assessors should also discuss the severity of any possible concerns and the potential solutions for these. All statements which have been scored 'No' will be considered for the severity of their impact on the species/individual, for the intensity/duration and reversibility of the impact and the need for urgent mitigation. As a result of this, a list of actions will be generated by this process and will be added to our Welfare Action Plan, allowing us to prioritise work which will promote best possible welfare across our collection. Sections should refer to this regularly as the action list will help them to prioritise the implementation of any actions required.

SCHEDULING WELFARE ASSESSMENTS

There are time constraints on achieving a complete audit of the vertebrate collection.

As such, assessments will be complete on the basis of capacity and of need. Species highlighted as having potential welfare challenges, through formal external inspections and through internal evaluations will be prioritized.

Individual assessments can also be used to assess quality of life when making end of life decisions.

Question no. in assessmen t	Category	Subcategor y	Question	Current guidance on how to respond to question	Suggested Evidence-base (being developed and piloted)
1	Nutrition		Clean fresh water is available on demand in a species appropriate manner to all animals	Environmental observations	
2	Nutrition		A diet sheet is available for the species and is reviewed incorporating best practice guidelines for nutrition and evidence-based literature as available	Review records and documentation	Presence of formal nutrition sheet
3	Nutrition		An appropriate quantity of high quality, nutritious food is available to all species/individuals and lifestages (include browse and/or graze where appropriate)	Environmental observations	
4	Nutrition		Food is presented appropriately for the species/individual (consider timing, method, position in enclosure, social interactions, variation)	Environmental observations	ZooMonitor behavioural+environmental data
5	Health		Body condition score is within normal limits	Animal condition scoring	Body condition records
6	Health		Regular weights are taken and recorded	Review records and documentation	ZIMS weight log or other weight log
7	Health		Animals appear to be in good physical condition (consider plumage/pelage/scales/scutes, skin, teeth, feet/hooves/claws, horns/antlers, eyes, nostrils etc.)	Animal condition scoring	
8	Health		Faeces are appropriately formed and normal for the species	Environmental observations	Consult record of faeces formation. Can be surveyed and logged with ZooMonitor.
9	Health		Animals have good muscle tone and fitness level with no evidence of weakness/debility	Animal condition scoring	Body condition and veterinary records

10	Health		Species/individuals are subject to preventative medicine regime (consider import testing, quarantine, vaccinations, parasitology, bacteriology etc)	Review records and documentation	
11	Health		Animals are free from signs of injury or pain (consider lameness, flinching, wincing, husbandry mutilations, current treatment etc)	Animal condition scoring	
12	Health		Animals are free from chronic or recurring acute disease/injury/impairment (and note remedial actions in place if not)	Review records and documentation	
13	Health		Veterinary care can be provided in a way which minimizes distress (consider operant conditioning, nets, darting, crush cages, separation areas)	Environmental observations	
14	Environmen t	Climate and lighting	Temperature levels/gradients are within parameters appropriate for the species year round (consider internal/external, seasonal, night-time, appropriate variation, choice, records, etc.)	Review records and documentation/ Environmental observations	Consult records of systematic, year-round measurements/records of temperature indoors and outdoors and compare to literature on comfortable range for the species.
15	Environmen t	Climate and lighting	Humidity levels/gradients are within parameters appropriate for the species year round (consider internal/external, seasonal, night-time, appropriate variation, choice, records etc.)	Review records and documentation/ Environmental observations	Consult records of systematic, year-round measurements of humidity levels indoors and outdoors and compare to literature on comfortable range for the species.

16	Environment	Climate and lighting	Light levels, quality and photo period are within parameters appropriate for the species year round (consider UV, photoperiod, flicker/glare, colour, internal/external, seasonal, night-time, appropriate variation, choice, records etc.)	Review records and documentation/ Environmental observations	Consult records of systematic, year-round measurements/records of light conditions indoors and outdoors and compare to literature on comfortable range for the species, taking into account visible spectrum of species and effects on circadian/circannual rhythms.
17	Environment	Climate and lighting	Noise and vibration levels are within parameters appropriate for the species year round (consider internal/external, seasonal, night-time, appropriate variation, choice, etc.)	Environmental observations	Consult records of systematic, year-round measurements of noise levels in different areas of the enclosure and near enclosure, including during special events. Then compare to literature on perceptible frequencies by the species and their comfortable range.
18	Environment	Enclosure design and features	Air quality is high and free from pollutants, heavy dust, aversive adours and is well ventilated	Environmental observations	
19	Environment	Enclosure design and features	Perimeter is secure (prevent escapes, excludes predators) and safe with no risk of harm (e.g. electric fences)	Environmental observations	
20	Environment	Enclosure design and features	The size, shape and topography of the enclosure is appropriate for the species to exercise, explore and exhibit normal territorial behaviours	Environmental observations	ZooMonitor behavioural+environmental data: enclosure use data

21	Environment	Enclosure design and features	Substrates are suitable for the species (consider locomotion (abrasion, traction, support); resting (comfort, depth, cleanliness); foraging (depth, cleanliness); burrowing (will support tunnels, depth, secure))	Environmental observations	ZooMonitor behavioural+environmental data: most used resting, foraging, burrowing sites, etc.
22	Environment	Enclosure design and features	Surfaces drain well with no pooling of faecal matter, urine or rainwater (unless this is appropriate to the species)	Environmental observations	
23	Environment	Enclosure design and features	Has appropriate shelters, retreats, visual barriers, off show areas from conspecifics and visitors	Environmental observations	ZooMonitor behavioural+environmental data: use of enclosure features for intended purposes
24	Environment	Enclosure design and features	Has appropriate shade and shelter from weather/climate	Environmental observations	ZooMonitor behavioural+environmental data: enclsoure use across different weather conditions
25	Environment	Enclosure design and features	Planting is appropriate for the species, providing shelter, shade, retreats, microclimate provision, feeding opportunities, and plants are not toxic and do not present an escape risk	Environmental observations	ZooMonitor behavioural+environmental data: use of enclosure features for intended purposes
26	Environment	Enclosure design and features	Furnishings allow appropriate species specific behavioural needs (climbing, swinging, jumping, perching, nesting, stretching, hiding, sleeping, flgiht, etc.)	Environmental observations	ZooMonitor behavioural+environmental data: behaviours displayed around furnishings
27	Environment	Enclosure design and features	There is evidence of changes/variability to the environment and furnishing (enrichment)	Environmental observations	
28	Husbandry		The animal has free choice over where it spends its time within the enclosure	Environmental observations	
29	Husbandry		The enclosure is clean and hygienic to the appropriate level for the species	Environmental observations	

30	Husbandry		The enclosure is not over-stocked	Environmental observations	
31	Husbandry		Social grouping is appropriate	Environmental observations	ZooMonitor behavioural data: social interactions
32	Husbandry		There is evidence of a pest control programme within the enclosure and no evidence (live animals, faeces etc) of pest species within the enclosure	Environmental observations	
33	Husbandry	Aquatic	Water feature is safe and of a depth/size/volume/gradient that allow species specific natural behaviours	Environmental observations	ZooMonitor behavioural+environmental data: behaviours displayed around water feature
34	Husbandry	Aquatic	Life support systems are fully operational and regularly maintained	Environmental observations	
35	Husbandry	Aquatic	Water flow and movement allows sufficient oxygenation	Environmental observations	Consult records of systematic/periodic measures of oxygen
36	Husbandry	Aquatic	Water testing is carried out on a routine basis and checked against acceptable ranges	Review records and documentation	
37	Behaviour		Animals can be observed approaching and moving away from conspecifics freely	Behavioural observations	ZooMonitor behavioural data: social interactions
38	Behaviour		Performs appropriate levels of self-care behaviours (grooming, preening, drinking, resting, comfort activities)	Behavioural observations	ZooMonitor behavioural data
39	Behaviour		Has mostly positive interactions with conspecifics or other animals	Behavioural observations	ZooMonitor behavioural data: social interactions
40	Behaviour		Has mostly positive or neutral interactions with staff/visitors	Behavioural observations	ZooMonitor behavioural data: social interactions (records interactions with staff and visiotrs)

41	Behaviour	Responds appropriately to novel changes in the environment (interest in appropriate enrichment vs fear/aversion/apathy)	Behavioural observations	ZooMonitor behavioural data
42	Behaviour	Can express choice and control over being in different (indoor/outdoor) areas (except for maintenance periods)	Behavioural observations	ZooMonitor behavioural data
43	Behaviour	Exhibit appropriate territorial behaviour (patrolling, scent marking)	Behavioural observations	ZooMonitor behavioural data
44	Behaviour	Exhibits appropriate foraging and feeding behaviours	Behavioural observations	ZooMonitor behavioural data
45	Behaviour	Exhibits play behaviour (alone or socially)	Behavioural observations	ZooMonitor behavioural data
46	Behaviour	Exhibit appropriate levels of rest and sleep	Behavioural observations	ZooMonitor behavioural data
47	Behaviour	No evidence of dysfunctional social interactions	Behavioural observations	ZooMonitor behavioural data: social interactions
48	Behaviour	No evidence of abnormal or stereotypic behavior	Behavioural observations	ZooMonitor behavioural data
49	Behaviour	Exhibits reproductive behaviours as appropriate to the species and individual (courtship, mating, nest-building, incubating, birth, rearing etc.)	Behavioural observations	ZooMonitor behavioural data Hormonal/physiological data
50	Behaviour	Exhibits species specific behavioural needs (rooting, burrowing, climbing, perching, social grooming etc.)	Behavioural observations	ZooMonitor behavioural data
